# Challenges and Opportunities for Centers on Aging: Part 2 - Sustainability and Leadership

**DOI:** 10.1093/geroni/igaf122.1037

**Published:** 2025-12-31

**Authors:** Debra Dobbs

## Abstract

With the increase in population aging there is a continued demand for training and research in the field of aging. With a new political landscape at state and national levels, there have been several challenges to Centers in Aging programs which have made growth and sustainability tenuous. This symposium highlights challenges facing institutions of higher education across the country as well as strategies and opportunities for program growth and development during times of uncertainty and fiscal constraint. The first paper focuses on sustainable partnerships between a private University in Massachusetts and State Departments on Aging to develop innovative, skill-based online training to build a competent and confident aging workforce. The second paper presents collaborative approaches being implemented at the Center for Successful Aging at a California State University to provide research and service opportunities to increase health and social service students’ knowledge and skills for working with an aging population. The third paper highlights strategies to sustain graduate research programs in a School of Aging Studies that is part of a large public University in Florida, heavily reliant on state and federal funds for their programs. The fourth paper highlights how aging centers can leverage their ability to conduct applied research as well as entrepreneurial initiatives to strengthen the value proposition of aging centers at a public University in Ohio. This symposium will serve as guidance for programs in the U.S. and other countries facing similar problems with sustainability of programs for educating future gerontologists. Directors of Aging Centers Interest Group Sponsored Symposium

